# Contracting Students for the Reduction of Foreign Language Classroom Anxiety: An Approach Nurturing Positive Mindsets and Behaviors

**DOI:** 10.3389/fpsyg.2020.01471

**Published:** 2020-07-14

**Authors:** Yinxing Jin, Lawrence Jun Zhang, Peter D. MacIntyre

**Affiliations:** ^1^School of Foreign Languages, Hainan Normal University, Haikou, China; ^2^Faculty of Education and Social Work, The University of Auckland, Auckland, New Zealand; ^3^Department of Psychology, Cape Breton University, Sydney, NS, Canada

**Keywords:** English, Chinese-first-language university students, contracting speaking, foreign language classroom anxiety intervention, positive mindsets and behaviors

## Abstract

The quasi-experimental study reported in this paper investigated whether contracting students’ speaking in the foreign language (FL) classroom could effectively mitigate their FL classroom anxiety. It also explored the working mechanisms of this approach to the reduction of classroom anxiety and examined the attitudes FL students had toward it. To these ends, 42 Chinese-as-the-first-language university students learning English as a foreign language (EFL) were recruited and placed into the experimental (*n* = 20) and comparison groups (*n* = 22). Both groups were tested for anxiety before and after completing a 1-week contract and a non-contracting treatment, respectively. The experimental group participants’ diaries were also collected, and their attitudes toward the intervention were elicited. Results showed that the experimental group’s level of anxiety decreased significantly more as compared with that of the comparison group, suggesting the better efficacy of contracting speaking in FL anxiety reduction. Diary analyses also suggested that contracting speaking could increase learners’ FL learning engagement; enhance their self-efficacy; facilitate their self-reflection of weaknesses and strengths as an FL learner; cultivate their character strengths and positive emotions; and diminish their fear, nervousness, and worries in class. Furthermore, the experimental group participants generally did not feel uncomfortable with the intervention. These findings were discussed in relation to classroom pedagogy for more effective delivery of FL education.

## Introduction

Foreign language (FL) anxiety has taken a central position in studies of emotion in the field of second language or FL teaching and learning. Empirical findings have shown that FL anxiety has the potential to disrupt behavior and interfere with interpersonal communication, cognition, and learning (Dewaele and MacIntyre, 2014; Castillejo, 2019). The adverse effects of anxiety can be damaging to a learner’s progress, if not properly handled (Zhang, 2000, 2001). Therefore, there is a need to explore interventions for anxiety reduction that are effective, easily applied, and psychologically acceptable to students in classroom contexts.

The present study addressed this research gap by using “contracting speaking” (that is, students sign a contract to commit to speaking in FL class) to attempt to reduce FL classroom anxiety. Anxiety can be considered a self-exacerbating syndrome where reactions to anxiety—such as distracting self-focused attention, worrying about making mistakes or appearing nervous to others, and avoiding practice opportunities that come with communication—help to maintain a feedback loop of that anxiety. By contracting speaking, we attempt to break the feedback among the causes and/or symptoms of anxiety. The research project explores what the participants thought about this approach using relevant diary entries. This kind of intervention has not been reported in the second language acquisition (SLA) literature as an anxiety-reducing technique.

A total of 42 Chinese-first-language university students in Year 2 were randomly assigned to an experimental or a comparison group in a 1-week intervention. The experimental group signed a formal contract, and the comparison group received an informal written instruction form. The rationale for this study lies in engagement theory that foregrounds the beneficial interactions among behavioral, cognitive, emotional, and social dimensions of engagement (Philp and Duchesne, 2016). More broadly, Oxford’s (2016) positive psychology model emphasizes a multidimensional view of learner well-being under the rubric of nine EMPATHICS dimensions (E = emotion and empathy; M = meaning and motivation; P = perseverance; A = agency and autonomy; T = time; H = hardiness and habits of mind; I = intelligences; C = character strengths; S = self factors such as self-efficacy, self-concept, self-esteem, and self-verification). The major goal of this study is to improve pedagogical practices through reducing learners’ negative emotions and boosting positive emotions.

## Literature Review

In an early synthesis of the literature on anxiety in language learning, Scovel (1978) revealed a confusing picture regarding the relationship between anxiety and FL achievement. Scovel hinted at a need to specify the anxieties relevant to FL learning and to develop corresponding measures. Horwitz et al. (1986) focused their attention on the construct of FL anxiety by defining it as “a distinct complex of self-perceptions, beliefs, feelings, and behaviors related to classroom language learning arising from the uniqueness of the language learning process” (p. 128). They also developed a 33-item Foreign Language Classroom Anxiety Scale (FLCAS) that has been used in a large number of studies (Horwitz, 2017). With the introduction of the FLCAS and other language-specific measures of anxiety, the literature has flourished and extended the conceptual boundaries of FL anxiety to include language use, processing of language at various stages, and skills-specific anxieties (e.g., MacIntyre and Gardner, 1994; Elkhafaifi, 2005; Gargalianou et al., 2016).

Research has shown that the effects of FL anxiety are pervasive, affecting learners at cognitive, academic, physiological, and social levels (MacIntyre, 2017). More specifically, anxiety arousal leads to increased self-related cognition (e.g., thoughts of failure and worry) and further consumes the cognitive resources needed for each of the three stages of FL learning: input, processing, and output (Eysenck, 1979; MacIntyre and Gardner, 1994). Academically, anxious students tend to perform worse in language proficiency or achievement tests than their more relaxed counterparts in part because anxiety tends to be associated with less effective study strategies, such as memorization (Gregersen et al., 2014). In addition, anxious students are less willing to engage in communication in FL with others, due in part to the self-deprecating worries about being able to communicate authentically and fear of the interlocutors’ negative evaluation (Sevinç and Backus, 2017; Dewaele, 2019). The physiological correlates of anxiety, including increased heart rate and skin conductance, suggest that it is a multidimensional experience that is only partially under the control of a learner (Gregersen et al., 2014; Sevinç, 2018). Horwitz et al. (1986) reported that anxious students showed many psycho-physiological symptoms that include tenseness, trembling, perspiring, heart palpitations, and sleep disturbances. When aroused, the physiological correlates of anxiety take time to settle down.

Foreign language anxiety affects learners’ verbal and non-verbal behaviors. MacIntyre and Gardner (1994) found that anxious students’ oral descriptions were less complex, were less fluent, and showed less of a native accent. Such patterns may be attributed to the reduced complexity and fluency in anxious students’ oral output, which may be connected to difficulties encountered in vocabulary learning and retrieval. Kim and Tracy-Ventura’s (2011) study showed that high-anxiety learners tended to be less accurate in the use of simple past tense. Thus, anxiety arousal in speaking may degrade three key indices of oral proficiency, that is, fluency, complexity, and accuracy. In addition to the verbal dimension of communication, Gregersen (2005) documented that anxious learners manifested differences in non-verbal behavior, including limited facial actions and brow behaviors but more eye-blink, than their more relaxed counterparts. Furthermore, the anxious learners smiled less often, maintained less eye contact with the teacher, exhibited a more rigid and closed body position, and used their hands less often for illustrative and regulatory speech-related purposes (e.g., adjusting clothing). In general, the findings support that communicative behaviors are restrained and rigid when anxiety arises and that elevated anxiety suppresses facial displays (Gregersen, 2005, p. 393).

If the effects of FL anxiety are complex, so too are its antecedents, which include a myriad of learner-internal and learner-external variables (Dewaele, 2017). MacIntyre (1999) and Dewaele (2013) reported that students who are predisposed to be anxious (i.e., trait anxiety) may transfer more anxiety into FL learning contexts. MacIntyre and Charos (1996) and Dewaele (2002, 2013) revealed that extroverts tend to have lower anxiety than introverts, although both extroverts and introverts have their unique strengths and weaknesses in developing language ability. In addition, dispositional positive orientation also predicts anxiety levels; students with higher positive orientation tend to hold a more positive conceptualization of self and others, and to perceive more social support, lowering FL anxiety levels (Jin and Dewaele, 2018). The role of positive attitudes toward self and others in lowering FL anxiety was further attested by Dewaele et al. (2018), who found that perceived FL competence and attitudes toward FL and FL teachers significantly negatively predicted students’ anxiety levels in learning the FL.

The complexity and pervasiveness of the sources and effects of FL anxiety, along with its rapid onset and marshaling of physiological processes, suggest that rather than eliminating it, the preferred goal of educators might be to ameliorate anxiety and reduce it to manageable levels (Dewaele and Al-Saraj, 2015). Many strategies have been tested by researchers for reducing learners’ FL anxiety levels. Nagahashi (2007) found that engaging in pair and group work in 12 classes taught by the researcher significantly decreased Japanese-first-language university students’ English classroom anxiety, suggesting that cooperative learning has an affective component (Early and Marshall, 2008). In addition, Dolean (2016) found that teaching songs in the classroom significantly reduced the anxiety level for one of the experimental groups. Galante (2018) reported a decrease of English classroom anxiety in 13 Brazilian adolescents after implementing a 4-month drama program. Thus, interventions that lead learners to approach language tasks in new ways or that produce alternative behavioral responses seem to carry the potential to reduce FL anxiety.

Anxiety reduction studies have also taken a positive psychology approach. Being a new psychological sub-discipline that emerged at the turn of this century, positive psychology concentrates on what makes people develop, flourish, and thrive, instead of zooming in on damages and pathologies (Seligman and Csikszentmihalyi, 2000). It has positive subjective experiences, positive individual traits, and positive institution as its three main pillars (Seligman and Csikszentmihalyi, 2000; Dewaele et al., 2019). Since positive psychology was introduced into SLA a few years ago (MacIntyre and Mercer, 2014), it has instigated a number of studies on positive emotions in SLA, particularly enjoyment (e.g., Jin and Zhang, 2018, 2019; Resnik and Schallmoser, 2019; Dewaele and Dewaele, 2020). More recently, researchers have attempted to investigate ways of anxiety reduction from a positive psychology perspective. Two such studies have been conducted to date, one by Gregersen et al. (unpublished) and the other by Jin et al. (unpublished).

Gregersen et al. (unpublished) tested the efficacy of using signature strengths in new ways for reducing writing anxiety in English as an FL. The participants were 45 Arabic-first-language university students, of whom 31 formed the experimental group and 13 the control group. The 31 experimental group students used their signature strengths in new ways while being involved in writing assignments nine times in a time frame of 3 weeks (three times per week), but the control group students remained untreated. The results showed that the two groups did not significantly differ in changes in writing anxiety, but the effect of the targeted intervention showed at the individual level. The researchers reported that anxiety in a female participant, Noor, decreased by nearly 30% after she applied her signature strengths of creativity, honesty, zest, and spirituality in English writing practices in new ways. She revealed in her self-narratives that participating in this study made her like her writing and feel optimistic, knowledgeable, and satisfied, contributing to her reduced anxiety.

Jin et al. (unpublished) found that reminiscing about English proficiency development significantly reduced Chinese-first-language university students’ anxiety in English class. Qualitative analysis into the reminiscing process showed that the participants reminisced about multiple linguistic and non-linguistic development and reported experiencing many positive emotions, outnumbering the negative ones approximately 3:1 in frequency, during the reminiscing process. The most prominent positive emotions were happiness, confidence, contentment, sense of accomplishment, pride, and enjoyment, but no negative emotion took precedence. Jin et al. (unpublished) claimed that it was the positive emotions and their savoring-generating functions that partially buffered the participants’ anxiety levels in English over time.

The present study tested the effectiveness of a type of anxiety reduction intervention—contracting speaking in FL class—originally suggested by Horwitz et al. (1986) but not tested in the FL anxiety literature. In this approach, students sign a contract to commit to speaking in FL class. Speaking is one of the key causes of anxiety, and lower willingness to communicate, one of its main effects (Horwitz et al., 1986; MacIntyre, 2017). The approach we took seamlessly dovetails with engagement theory that highlights the beneficial interaction between behavioral and emotional engagement (Philp and Duchesne, 2016). Speaking with another learner integrates the multiple dimensions of engagement, with particular focus on the social aspect of listening to each other. However, speaking implicates more than just learner engagement. Oxford’s (2016) EMPATHICS framework highlights a diverse collection of processes within its nine dimensions. EMPATHICS is a model for language learners’ psychological well-being in which Oxford emphasizes that negative emotions such as anxiety fit within a larger, dynamic system reflecting the psychology of the learner. Perhaps the key implication of this framework is that engaging with activities that target one part of the system, as we are proposing here with contracting speaking as anxiety reduction, will draw upon and potentially affect resources in other parts of the system (e.g., positive emotion, motivation, and the self). By willingly signing a contract to speak more often in the classroom, and abiding by its terms, learners simultaneously are expressing their motivation, perseverance, sense of agency, autonomy, and responsibility to their classmates, who are doing the same. Empirical studies showed that interventions that facilitate oral use of language led to lower FL anxiety in the classroom (e.g., Dolean, 2016; Galante, 2018). EMPATHICS emphasizes that contracting speaking will draw upon learners’ psychological resources and, if it works, carry implications beyond the task at hand, such as reducing anxiety and promoting a greater sense of well-being.

Specifically, this study aimed to answer the following questions:

1. Does contracting speaking in FL class significantly reduce learners’ FL classroom anxiety levels?
2. What do students say about this behavioral approach and its effects?

## Methodology

### Research Design

This study adopted a 2 × 2 study design, with an experimental group and a comparison group, each being tested for FL anxiety levels before and after the intervention. With this research design, we are able to investigate contracting speaking as an alternative approach to FL anxiety reduction.

This study also elicited the experimental group participants’ attitudes toward the intervention. The rationale resides in our intention to assess its acceptance by learners and possible unintended side effects. Moreover, these students’ diary entries recorded on each intervention day were collected to take advantage of the student as “…a crucial witness of his or her own learning process” (Dewaele, 2005, p. 369). These diary entries thus provided detailed accounts of the students’ responses to the intervention.

### Participants

Forty-eight university-level English major students in Year 2 who were recruited from a public university in South China formed the initial sample pool (24 participants in both experimental and comparison groups). The final sample size constituted 20 students for the experimental group and 22 for the comparison group because three individuals dropped out and three were disqualified for failing to implement the intervention. All the remaining 42 students were young women, reflecting the fact that the majority of students in Chinese universities’ English programs are female. The average age was 19.68 years (*SD* = 2.39) for the experimental group and 19.94 years (*SD* = 0.77) for the comparison group. Most of the participants started learning English in primary school, with few students beginning during junior high school.

Generally, students in both groups had learned English for close to 10 years by the time of data collection: *M* = 10.15 years, *SD* = 2.10 for the experimental group; *M* = 10.07 years, *SD* = 1.62 for the comparison group. All the participants were considered at an intermediate level in English proficiency. As required by their program, these students were taking several English courses such as *Integrated English, British Literature*, *English Writing*, and *English–Chinese Translation*, taught by different teachers. They were also learning one of German, Japanese, Spanish, or Korean as a third FL.

### Instruments

#### The English Classroom Anxiety Scale

The English Classroom Anxiety Scale (ECAS) is a five-point Likert scale in Chinese developed from Horwitz et al.’s (1986) FLCAS through exploratory and confirmatory factor analysis (Jin et al., unpublished) and contains seven positively worded items and four negatively worded items rated on: *strongly disagree* (1), *disagree* (2), *neither agree nor disagree* (3), *agree* (4), and *strongly agree* (5). The negatively worded items were reversed-scored. The total obtainable scores on the ECAS range from 11 to 55, with higher scores representing higher levels of English classroom anxiety.

Jin et al. (unpublished) reported a Cronbach’s alpha of 0.84 and 0.86 at two time points over 1 month for the ECAS, almost the same as the present study (0.83 and 0.86). The test–retest reliability coefficients in the two studies for the scale were also similar: *r* = 0.82 *p* < 0.001 in the control group in Jin et al. (unpublished) and *r* = 0.76 in the comparison group of this study. In addition, Jin et al. (unpublished) found that the ECAS had satisfying criterion validity. It was more closely related to FL anxiety scales (correlation ranged from 0.59 to 0.73, all *p* < 0.001, with FL reading, listening, writing, and speaking anxiety scales) but less related to trait anxiety measures: *r*_*s*_ = 0.49, *p* < 0.005, with a test anxiety scale, and *r* = 0.09, *p* = 0.59, with a trait anxiety scale.

#### Attitude Check Toward Contracting Speaking

This attitude check consisted of one item asking about the participants’ degree of comfort toward the contracting intervention. The anchors for this item were *strongly disagree* (1), *disagree* (2), *neither agree nor disagree* (3), *agree* (4), and *strongly agree* (5). The more a student agreed to this item, the more comfortable she or he felt about the contracting intervention.

#### The Contract for the Experimental Group

The contract for the experimental group contained two parts. The first part (Appendix A) spelt out who would carry out the contract in which English courses and what the contract conditions were. The period of time when the contract was in force was also specified. The second part (Appendix B) was a procedural checklist through which the participants reported their contract compliance, allowing self-monitoring and self-management (Hawkins et al., 2011).

#### The Informal Written Form for the Comparison Group

The students in the comparison group received a written form with instructions requesting these students to volunteer speaking in English class and try to freely, clearly, and comprehensively express themselves with no worries about others’ negative evaluation when being called on by teachers. This written form was not signed by any party and was not provided with a procedural checklist.

### Procedures

The first author called for volunteers for this study in his teaching sessions by explaining to the targeted students that this study was concerned with English learning emotion and that participating in the study would benefit their English learning. One hundred and eight students responded positively. These students filled out the EACS and then listed three class activities about which they felt fairly anxious. In the end, 48 students who reported that speaking in English was a great concern were enrolled. To subgroup the participants, we asked a non-English-major student who was totally blind to the research purpose to randomly choose 24 numbers from 48 numbers representing all the participants in this study. The 24 students chosen formed the experimental group, who were contracted to speak in any English classes during the intervention period, and the remaining 24 students, the comparison group.

The first author met both groups separately in two sessions a few days after the participation invitation procedure. In Session 1, the first author met the experimental group students, who were first required to write down a number indicating the maximum number of times they could volunteer speaking in English class each day in the coming week. The contract was then distributed and explained. After that, the participants registered the number they just wrote down in the contract with no changes being allowed. They were also reminded to keep diaries each day to record the changes that they thought implementing the contract brought about. In the end, the contract was signed and fingerprinted by the researcher and the participants because “using a formal looking contract form adds to the student’s perception of the importance of the contract” (Strahun et al., 2013, p. 4). The participants kept their contracts until the intervention was over. Immediately following Session 1, the first author met the comparison group students, and the written form was distributed. No diary recording was required from these students. The researchers did not divulge one group’s intervention to the other. No students out of this study were informed of the intervention for either group, and neither were teachers besides the first author.

Seven working days later, the researcher met the two groups separately and consecutively again. Both groups filled out the ECAS for a second time. The experimental group students also returned their contracts and handed in their diaries.

### Data Analysis

Major data analyses proceeded in three steps. The first step was to investigate the changes in anxiety for both the experimental and comparison groups (Question 1), using a mixed ANOVA. The second step was a descriptive analysis conducted on the single-item attitude scale data to see whether the participants felt comfortable about the contracting intervention (Question 2).

The third step was to sort the diary materials into themes to provide insights into the ways in which contracting speaking affected the students (Question 2), referring to the grounded theory method (Charmaz, 2006). More specifically, we first coded line by line the participants’ responses to contracting speaking, during which we accepted the participants’ terms as “vivo codes” (Charmaz, 2006, p. 55) to preserve the participants’ voices, and more often, we created terms to code the participants’ responses to best represent their meaning. Following that, the codes with the same properties were integrated into categories. For instance, actively preparing for class and in-class involvement were merged into increased engagement in FL learning because both of them featured an increased focus on language learning.

The categorization process was iterative, including coding and recoding responses as well as merging and dismantling categories as a result of constantly comparing codes and categories. Once this process came to an end, the findings and raw materials were given to a Chinese researcher with a Master’s degree in applied linguistics to ensure that no relevant responses were missed and that responses were sorted into proper categories, which in turn were integrated appropriately. The first author discussed with this researcher to resolve any discrepancies that occurred between them.

## Results

### Overall Anxiety Changes Over Time

Within a week, one student implemented the contract 3 times, two students, 4 times, and the remaining students, 5 to 11 times, suggesting that the contracts were taken seriously by the learners. Scores on the attitude check item indicated that most students expressed some degree of comfort with the contracting process; only 4 of the 24 learners (<17%) in the experimental group found the intervention uncomfortable. A 2 × 2 mixed model ANOVA was performed to evaluate overall anxiety changes over the week for the between-groups factor (experimental versus comparison groups). Time was the within-subjects variable, with two levels (pre- and posttests). Exploring the data showed that the ECAS scores for both groups at two time points were normally distributed as suggested by non-significant Shapiro–Wilk tests. Levene’s test for homogeneity of variances showed that the experimental and comparison groups had similar levels of variability for both the pretest [Levene’s *F* (1, 40) = 0.36, *p* = 0.55] and posttest [Levene’s *F* (1, 40) = 0.16, *p* = 0.69]. Mauchly’s test of sphericity for the repeated measures factor could not be computed, as there were only two testing occasions.

**FIGURE 1:**
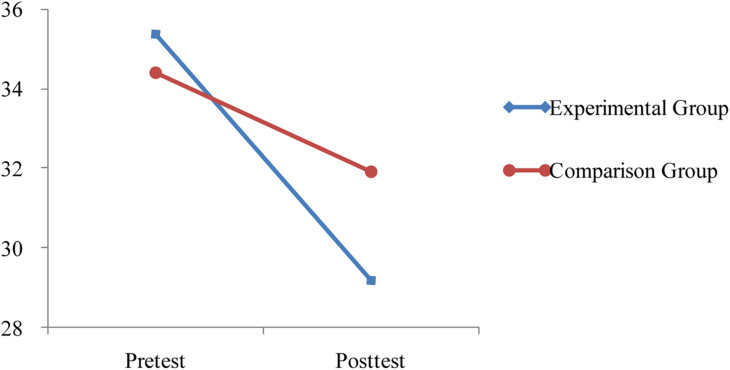
The time × group interaction.

The results showed that there was a non-significant main effect for group *F* (1, 40) = 0.25, *p* = 0.62, but a significant main effect of time on anxiety levels, *F* (1, 40) = 28.92, *p* < 0.001. More importantly, the main effects were superseded by a significant time × group interaction effect, *F* (1, 40) = 5.23, *p* = 0.03. Although anxiety level decreased in both the experimental and comparison groups over the intervention period, the amount of change significantly differed in the two groups. The experimental group (Time 1: *M* = 35.40, *SD* = 5.89; Time 2: *M* = 29.20, *SD* = 6.74) showed more of a decline in anxiety than the comparison group (Time 1: *M* = 34.41, *SD* = 6.44; Time 2: *M* = 31.91, *SD* = 5.44). This interaction effect is plotted in Figure 1.

### Findings of Diary Analyses

The diaries contained insightful contents to understand how the contract executed in this study functioned in FL anxiety. These contents were sorted into themes reported in this section. Diary excerpts were provided (see the italicized text with the initials of the diarists) as a support to the themes. A total of five inter-related themes were identified, suggesting that the contracting process created a complex set of adaptations by the learners, many of which are typically associated with enhanced learning outcomes and reduced anxieties. The specific themes include increased engagement, self-efficacy, self-reflection, reduced fear, and cultivating both character strengths and positive emotions.

#### Increasing Engagement in FL Learning

The increased engagement in FL learning occurred both inside and outside classroom time. Students tried to prepare for class well before it started and carried on learning tasks that were performed in class. The in-class behaviors included: thinking hard, volunteering to answer questions, competing with classmates for a chance to speak, and concentrating on what was taught. Representative diary excerpts are as follows:

*In order to perform the contract well, I was more attentive in class than ever before. In the first dozens of seconds when the teacher set out to explain a translation, I thought hard the correct answer and would raise my hand, provided I was sure about the answer* (QYH).

*As the class just started, I raised my hand to answer the teacher’s first question because I was eager to finish the contracted tasks* (LY).

*Implementing contract made me more serious-minded and less distracted in class* (WJ).

#### Enhancing Self-Efficacy

The diaries witnessed a growth of self-efficacy in some students. That was attributed to factors like voluntarily making an oral report, practicing speaking frequently, obtaining opportunities to show oneself in the presence of others, gaining new knowledge, receiving useful advice from the teacher, wining teacher recognition, and encountering unexpected success and making a breakthrough in English learning. Two factors were referred to more often, which were successfully performing a speaking task and teacher recognition. The excerpt below was adopted from QYH’s Day 3 diary.

*…I did not feel nervous any more owing to speaking frequently these days. I also did not care about what other people thought of me and did not fear to lose face. I felt that I was no longer a silent student and that I won my teacher’s recognition*.

#### Enhancing Self-Reflection

Being involved in the survey created opportunities for students to reflect on their weaknesses as FL learners, particularly insufficient English skills and inappropriate English learning strategies. For example, QYH noted that her English pronunciation was not good. LY realized that unwillingness to answer questions slowed down her progress in English learning and did not help her to know which aspects of her English should be improved. GSS believed that the lack of listening training partially led to her poor speaking ability. For the reasons why she had no confidence in class and why she was not able to answer questions, GSS concluded: *“The reasons might be that I did not have solid basic knowledge and did not contemplate the teacher’s question.”*

Students also noticed their strengths and proposed adequate learning strategies. Several individuals felt that they were capable English learners. LY thought that preparing for English class sufficiently and thinking carefully about the teacher’s questions even when not being called on would help with anxiety reduction. She also agreed that students should make peace with imperfect answers. WM revealed that voluntary answering led to improvement of oral ability, built a deeper impression on the issues addressed, helped to win the teacher’s and classmates’ recognition, fueled learning enthusiasm, and forged courage and confidence. GMM reflected that slowing down speech rate would contribute to fluency.

#### Diminishing Fear, Nervousness, and Worry

Students’ fear and nervousness were alleviated once they prepared for class in advance, actively sought chances of speaking in class, got accustomed to speaking, received others’ acknowledgments, and gained self-efficacy. Further, fear or nervousness was shared by class members when they jointly created an atmosphere featuring deep engagement. Two example excerpts are shown below:

*I spoke more naturally in class over a week and thus did not feel as tense as before. I noticed that a majority of students who participated in the survey talked actively, fluently, and naturally. It meant that we all made progress*.

*Fortunately, the presentation was good. My teacher highly appreciated it. I felt all my efforts that had been taken were worthwhile. With this experience of presenting alone, I thought I would not be nervous any more. I had been fearful of this teacher before because I did not know what she thought of me. Due to this experience, my fear was relieved*.

Worry about others’ evaluation causes anxiety, especially when students think that their performance would be judged as poor. However, a student, CJW, noted that she would not worry about what peer classmates think of her answer after a successful voluntary attempt to answer a question that led to the teacher’s affirmation. After multiple practices in class, LY expressed, *“My heart did not beat as fast as before and I did not consider all kinds of consequences now when raising hand to answer questions.”*

#### Cultivating Character Strengths and Positive Emotions

Students developed hope in the process of implementing their contract. They wished that they would become more active in English class (LY), improve their English proficiency (SN), and overcome psychological barriers (SN). Being hopeful, students were more loyal to FL class (YXL and GSS).

The diary materials also showed that contracting speaking cultivated courage. As aforementioned, students became less afraid of speaking and/or less worried about others’ judgment. Several reported that they made a breakthrough in English class. As the intervention was coming to a close, WLQ, for whom voluntarily answering questions was quite difficult, nevertheless stood up, responding to her teacher’s question, and WJ did her first voluntary speaking in translation class.

Positive emotions about English learning were forged. Owing to the teacher’s compliments arising from their successfully performing a speaking task, WJ and GSS felt happy or more enthusiastic about English learning. CX and WLQ developed interest in or enjoyment of English class. In addition, students also might have felt proud when they successfully responded to a question or won others’ admiration.

## Discussion

The frequently reported negative effects of FL anxiety on FL learning in the literature call for studies of FL anxiety reduction. The present study explored the efficacy of contracting speaking in FL class in reducing learners’ FL classroom anxiety. Evidence from the results of the mixed ANOVA revealed a significant interaction between time and group. The experimental group demonstrated significantly more anxiety reduction than the comparison group. This finding confirmed Horwitz et al.’s (1986) hypothesis that contracting interventions might be useful in anxiety reduction.

The procedures used in this study seemed to generate the desired commitment from the students through implicit reinforcements. First, signing and fingerprinting of the speaking contract by the researcher and the participants created a sense of importance of the contract, increasing the participants’ commitment to it. Second, asking the participants to fill in the procedural checklist to record their contract compliance and to write in a diary pushed these students to stick to the contract terms. Nevertheless, we suggest that teaching practitioners should be cautious in using any types of reinforcements when they aim at managing their students’ emotions, because reinforcements may add to the students’ emotional loads, affecting the power of an intervention.

An attitude check score showed that the experimental group participants as a whole felt comfortable with the intervention. We must take note, however, of the four students who reported that they did not like the contract. Such interventions are unlikely to produce uniform reactions across a group of learners—some students welcome it, but some others do not like it. The findings also remind researchers to focus their attention on not only average scores but also on individuals’ responses, because individual differences disappear in group means (Dewaele, 2005). Combining the findings of the mixed ANOVA and attitudes toward the intervention suggests that contracting speaking is feasible and, on balance, reduces FL anxiety more than not having the intervention, though not every student embraces the approach.

Diary entries provided useful information to understand the processes by which the contracting intervention affected the participants’ anxiety levels. The contracting intervention was shown to have the potential to cultivate learners’ character strengths and positive emotions such as hope, courage, enjoyment, interest, and pride. These findings reflect the existing theoretical or empirical research into the effect of motivation (Piniel and Csizér, 2015), self-efficacy (Dewaele et al., 2019), knowledge of learning strategies (Tran and Moni, 2015), character strengths (Jin and Dewaele, 2018), and positive emotions (Dewaele and MacIntyre, 2014) on FL anxiety. In addition, diary keeping itself gave the participants a second chance to reflect on their linguistic and non-linguistic gains during the process of performing intervention tasks, strengthening their positive experiences associated with this process, which led to positive self-cognition and, in turn, the decrease of anxiety. The finding supports MacIntyre and Gardner’s (1991), Jin et al’s. (unpublished), and Gregersen et al. (unpublished) findings that a focused essay on positive experiences could lead to the reduction of FL anxiety.

The diaries suggested that contracting speaking produced complex effects that extended to multiple parts of the learners’ psychological systems. Learners reported increased behavioral engagement with FL learning, which was associated with increased positive emotional engagement (enthusiasm, enjoyment, interest, and pride) and decreased negative emotional engagement (anxiety, fear, worry, and nervousness), contributing to the learners’ overall emotional well-being (Oxford, 2016; Philp and Duchesne, 2016). Many of the dimensions in Oxford’s EMPATHICS framework were activated by the contract as well. Due to the binding power of the contract, the participants persisted through the initial difficulties in implementing the contract, building their perseverance, which Oxford (2016) says is one of the key elements in language learning motivation. The character strengths of courage and hope grew in the participants when they became less afraid of FL class or made a breakthrough in FL class. The self-related dimensions of EMPATHICS also became more salient to the learners as they increased in self-efficacy with successful speaking opportunities and positive reactions from the teacher (better relationships) and self-verification through reflecting on their own strengths.

It must be emphasized that the intervention produced these effects over a short time in a well-established system. Particularly at the initial stage of contract implementation, some students felt pressure to change from a silent student to an active player in the classroom. Classroom behaviors that are long-term habits may be linked to one’s personality and cultural background. Consequently, some students might feel anxious for not immediately plunging into contract implementation, be worried about completely failing to fulfill the contract in the end, or be scared of being criticized by the researcher, who was also their teacher. At the end of the contract term, one student expressed regret that she did not follow the contract instructions well. This sense of failure might result in unintended feelings of shame or regret. Yet the majority of learners reported some degree of comfort with the contracting intervention agreed to in a social setting, possibly because it took the focus away from the self as the origin of speaking in class and put the emphasis on fulfilling the obligations of a contract. The contract appeared to increase cognitive, emotional, behavioral, and social forms of engagement among the study participants (Philp and Duchesne, 2016).

The limitations of the study must be noted. First, the participants were English majors who were internally motivated to perform the interventions. It is unknown whether the interventions, particularly the non-contracting intervention, would work well among non-English-major students. Secondly, we did not systematically observe the English classes that the experimental group students attended, which restricted a deeper understanding of the changes that the targeted intervention brought about for the participants. Third, all participants were females. The little available evidence shows that gender does not necessarily affect results of behaviorally contracting interventions (Bowman-Perrott et al., 2015). Yet the effectiveness of the current interventions on male students remains a question. Fourth, the contract period only lasted for 1 week, which is a short time span, so it might be that the experimental group students’ anxiety was suspended only for the intervention time. However, the pretest–posttest study design did not allow us to address longer-term patterns of anxiety change, or possible rebound. Thus, this study cannot tell how long the effect of the intervention of interest on FL anxiety levels might last.

## Conclusion

This study provides evidence that contracting speaking is a feasible approach to FL anxiety reduction over a short period of time and that students generally feel positively toward the approach. Future research is needed to test the effectiveness of this technique in other contexts, including contracts that can be used to deal with skills-specific FL anxiety (such as writing and listening anxiety). In addition, the roles of contracting interventions in nurturing FL learning-related character strengths and positive emotions also deserve an explicit investigation, given the findings regarding hope, courage, enjoyment, and interest in this study. A great body of evidence has suggested the importance of positive emotions for learning and living. Thus, seeking ways to enhance students’ positive emotions in FL learning is an essential part of second language acquisition research.

## Data Availability Statement

The datasets generated for this study are available on request to the corresponding author.

## Ethics Statement

This study involving human participants was reviewed and approved by an ethics committee organized by the School of Foreign Languages, Hainan Normal University, China. All the participants voluntarily took part in this study. The experimental group students provided their written informed consent to participate in intervention by signing and fingerprinting the contract.

## Author Contributions

YJ and LZ discussed and designed the study. YJ collected the data and drafted the manuscript. YJ, LZ, and PM revised it for submission. All authors contributed to the article and approved the submitted version.

## Conflict of Interest

The authors declare that the research was conducted in the absence of any commercial or financial relationships that could be construed as a potential conflict of interest.

## Appendix A

### The Contract for the Experimental Group

**Contract**

**The Parties to the Contract**

**Party A:** *Teacher Name*

**Party B:** *Student Name*

**Contract Terms**

As negotiated with Party A, Party B is committed to performing the tasks that are described in Terms 1 and 2 in English classes for courses as listed below from _____ to _____ (time range) as well as those tasks in Terms 3 and 4:

1. _____ times of voluntary speaking in Chinese or English each day.
2. In the case of being called on by teachers, Party B will try his or her best to freely, clearly, and comprehensively express his or her own views with no worries about whether peer classmates or teachers would negatively evaluate his or her speaking.
3. Filling in the *Procedural Checklist for Contract Compliance* on Page 2 every day to document how well Terms 1 and 2 are followed.
4. Keeping a diary every day to elaborately record the consequences of performing Terms 1 and 2 (e.g., the perceived changes in self-efficacy, teacher or peer students’ attitudes toward oneself, oral ability, and English learning enthusiasm).

**Table:**

**English Courses**	
Signature and Fingerprint	Signature and Fingerprint
(Party A)	(Party B)
Date	Date

### Sample Procedural Checklist for Contract Compliance

**Table:**

Date	Compliance
**Nov. 28 (Monday)**	Please check (√) appropriate □ to indicate how many times you complied with Terms 1 & 2. **Term 1 Compliance**:0□; 1□; 2□; 3□ Time 1: Course:____________; What did you say?___________________ Time 2: Course:____________; What did you say?___________________ **Term 2 Compliance**:0□; 1□; 2□; 3□ Time 1: Course:____________; What did you say?___________________ Time 2: Course:____________; What did you say?___________________
